# A Systematic Review of the Botanical, Phytochemical and Pharmacological Profile of *Dracaena cochinchinensis*, a Plant Source of the Ethnomedicine “Dragon’s Blood”

**DOI:** 10.3390/molecules190710650

**Published:** 2014-07-22

**Authors:** Jia-Yi Fan, Tao Yi, Chui-Mei Sze-To, Lin Zhu, Wan-Ling Peng, Ya-Zhou Zhang, Zhong-Zhen Zhao, Hu-Biao Chen

**Affiliations:** School of Chinese Medicine, Hong Kong Baptist University, 7 Baptist University Road, Kowloon, Hong Kong, China

**Keywords:** *Dracaena cochinchinenesis*, Dragon’s blood, botanical source, phytochemistry, pharmacological activity, clinical application

## Abstract

“Dragon’s blood” is the name given to a deep red resin obtained from a variety of plant sources. The resin extracted from stems of *Dracaena cochinchinensis* is one such source of “dragon’s blood”. It has a reputation for facilitating blood circulation and dispersing blood stasis. In traditional Chinese medicine, this resinous medicine is commonly prescribed to invigorate blood circulation for the treatment of traumatic injuries, blood stasis and pain. Modern pharmacological studies have found that this resinous medicine has anti-bacterial, anti-spasmodic, anti-inflammatory, analgesic, anti-diabetic, and anti-tumor activities, while it is also known to enhance immune function, promote skin repair, stop bleeding and enhance blood circulation. Various compounds have been isolated from the plant, including loureirin A, loureirin B, loureirin C, cochinchinenin, socotrin-4'-ol, 4',7-dihydroxyflavan, 4-methylcholest-7-ene-3-ol, ethylparaben, resveratrol, and hydroxyphenol. The present review summarizes current knowledge concerning the botany, phytochemistry, pharmacological effects, toxicology studies and clinical applications of this resinous medicine as derived from *D. cochinchinenesis*.

## 1. Botanical Source

### 1.1. Relationship between D. Cochinchinensis and Dragon’s Blood

Dragon’s blood is a red resin obtained from different species of four distinct plant genera: *Croton, Dracaena, Daemonorops, and Pterocarpus*. In Chinese folk medicine the resin extracted from stems of *Dracaena cochinchinensis* has mainly been used as dragon’s blood [1]. In this review, we thus focus on the dragon’s blood derived from *D. cochinchinensis*.

### 1.2. Plant Occurrence

*D. cochinchinenesis* is exclusively distributed in China (southern Yunnan and Guangxi provinces), Vietnam and Laos, and it has been listed as a national endangered plant since 1987 [2]. This plant is generally found on sunny cliffs in limestone areas of steep mountains, typically at elevations of 1,300–1,700 m [3]. Botanists estimate there are only about 200,000 plants living in the wild, moreover, the wild resource of *D. cochinchinenesis* is becoming increasingly scarce due to excessive collection.

### 1.3. Botanical Description

**Figure 1:**
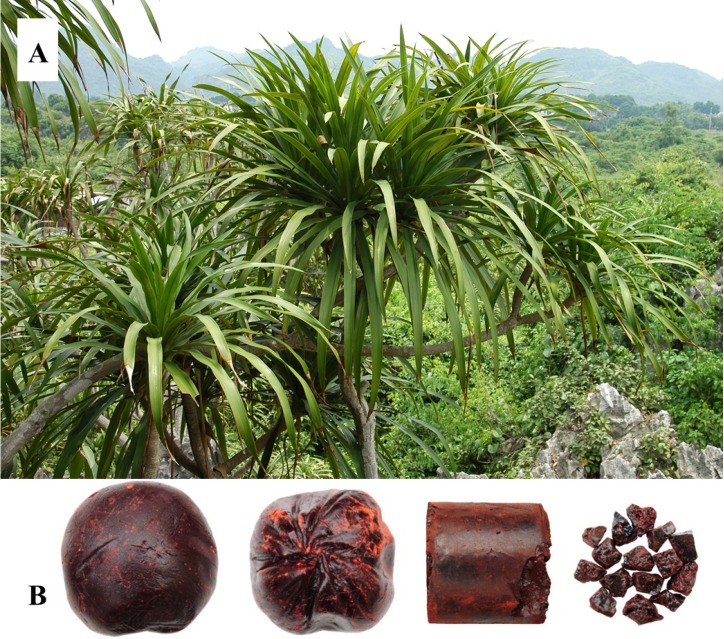
Photos of *Dracaena cochinchinensis* plant (**A**) and “Dragon’s Blood” medicinal materials (**B**).

Evergreen trees, 5–15 m tall. Stem thick, multi-branched, bark grayish white, smooth, becoming grayish brown when old, flake-shaped abscission, young branches with annular cicatrices. Leaves crowded at the apex of branches, overlaying each other, sword-shaped, thinly coriaceous, 50–100 cm long, 2–5 cm wide, amplexicaul, sessile. Flowers in panicles, each panicle more than 40 cm long, rachis densely papillose-pubescent, more flourished when young. Flowers in clusters of 2–5, oyster white; pedicels 3–6 mm long, nodes located near the top; tepals 6–8 mm long, about 1/4–1/5 of lower part combined; filaments flat, 0.6 mm wide, upper part with red-brown warts; anther 1.2 mm long; style slender. Berry spherical, 0.8–1.2 cm in diam., 1–3-seeded. Flowers appear in March, followed by fruit from July to August [2]. The photos of the plant and the medicinal materials are showed in Figure 1 [4].

### 1.4. The Formation of Dragon’s Blood in D. Cochinchinensis

As *D. cochinchinensis* grows, particularly in response to mechanical damage or insect infestation, resin accumulates in xylem cells in the hollow pith of old stems. The xylem gradually hardens. Resin rich in its purple xylem is extracted with 95% alcohol, then the resin that accounts for 32% of the wood can be extracted, and can be used directly as the Chinese ethnomedicine dragon’s blood [5]. Recently, researchers have reported that the production of the dragon’s blood occurs mainly via the phenylalanine ammonia-lyase (PAL) pathway. Also, the wounding plus causal fungal infection and keeping the wound moist are essential for efficient dragon’s blood formation in *D. cochinchinensis*. Fungi like *Fusarium proliferatum*, and chemicals such as oxalic acid and leucine can increase the yield of dragon’s blood formation [6,7,8].

### 1.5. Quality Control of Dragon’s Blood

Dragon’s blood appears in the market as irregularly shaped blocks; its morphological characteristics are as follows: deep purple surface, lustrous with local red dust adhesion. Hard, brittle, and the cross-section is smooth. Glass-like luster, odorless; slightly astringent in taste, and sticky when chewed. According to the Chinese national quality standard WS3-082(Z-016)-99(Z) for dragon’s blood, any sample should contain not less than 0.4% of loureirin B (C_18_H_20_O_5_), calculated with reference to the total weight.

## 2. Phytochemistry

Flavonoids are the main chemical constituents of dragon’s blood. Terpenes, steroids, saponins and phenols have also been identified as constituents.

### 2.1. Flavonoids

#### 2.1.1. Chalcones and Dihydrochalcones

**Figure 2:**
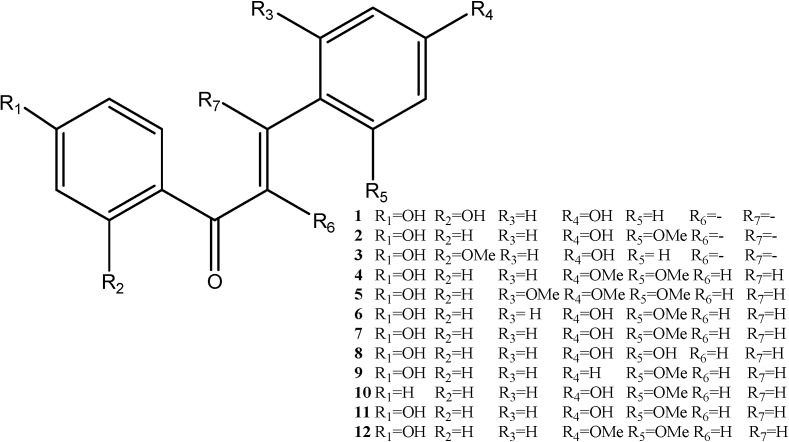
Structures of chalcones and dihydrochalcones.

Chalcones and dihydrochalcones are abundant in dragon’s blood. In general, most of the dihydrochalcones are substituted in the 4,4'-position. Some are substituted in the 2,6-position, and a few are substituted in the 2,4,6-position. Most of the dihydrochalcones obtained from the original plant have free phenolic hydroxyl groups. When the red resins form, the percentage of methylated ingredients increases significantly. The compounds that can be isolated from dragon’s blood include [9,10] chalcones such as 2,4,4'-trihydroxychalcone (**1**), 2'-methoxy-4,4'-dihydroxychalcone (**2**) and 2-methoxy- 4,4'-dihydroxychalcone (**3**) and dihydrochalcones like loureirin A (**4**), loureirin B (**5**), loureirin C (**6**), loureirin D (**7**), 2,4,4'-trihydroxydihydrochalcone (**8**), cochinchinenin A (**9**), 4-hydroxy-2-methoxy-dihydrochalcone (**10**), 4,4'-dihydroxy 2,6-methoxydihydrochalcone (**11**), 4'-dihydroxy-4,6-dimethoxy- dihydrochalcone (**12**). The corresponding structures are depicted in Figure 2.

#### 2.1.2. Flavanones and Flavans

**Figure 3:**
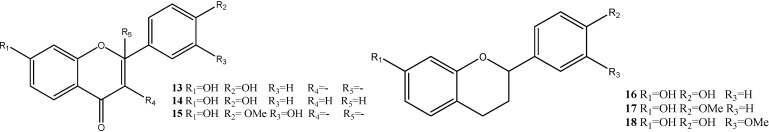
Structures of flavanones and flavans.

There are few flavanones and flavans in *D. cochinchinensis*. They include 7,4'-dihydroxyflavone (**13**), 7,4'-dihydroxy-dihydroflavone (**14**), 4'-methoxy-3'7-dihydroxyflavone (**15**), 4',7-dihydroxy- flavan (**16**), 7-hydroxy-4'-methoxyflavane (**17**), and 4',7-dihydroxy-3'-methoxyflavan (**18**) [11,12]. The structures are shown Figure 3.

#### 2.1.3. Polymeric Flavonoids

**Figure 4:**
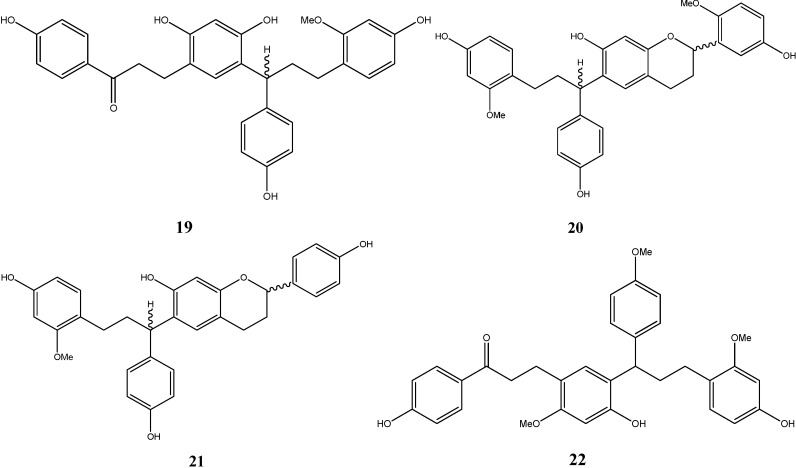
Structures of polymeric flavonoids.

Zhou *et al.* isolated three polymeric flavonoids from dragon’s blood, namely cochinchinenin (**19**), 2'-methoxysocotrin-5'-ol (**20**), socotrin-4'-ol (**21**), and cochinchinenin C (**22**) [12,13]. Their structures are depicted in Figure 4.

#### 2.1.4. Chromogen Ketones

**Figure 5:**
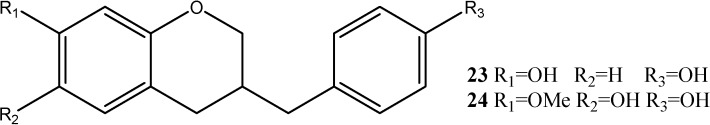
Structures of chromogen ketones.

7-Hydroxy-(4-hydroxybhenyl) chromane (**23**) and 6-hydroxy-7-mrthoxy-3-(4'-hydroxybhenyl) chromane (**24**) have been isolated from dragon’s blood [12,13]. The structures are shown in Figure 5.

### 2.2. Terpenes, Steroids and Steroidal Saponins

**Figure 6:**
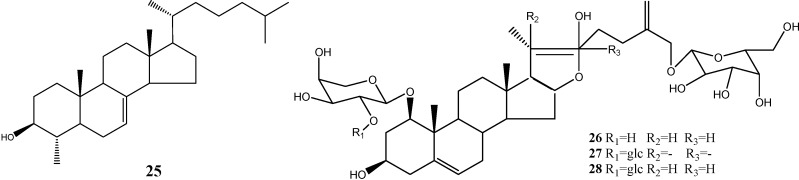
Structures of terpenes, steroids and steroidal saponins.

Steroids and triterpenoids are widely distributed in Nature. 4-Methylcholest-7-ene-3-ol (**25**) was isolated from dragon’s blood from Guang Xi Province [13]. Other compounds were isolated from dragon’s blood from Yun Nan Province, including 26-*O*-β-d-glucopyranosyl-furostanol-5,25(27)- diene-1β,3β,22β,26-tetraol 1-*O*-α-l-arabinopyranoside (**26**), 26-*O*-β-d-glucopyranosylfurostanol-5,20 (22),25(27)-triene-1β,3β,26-triol-1-*O*-[α-l-rhamnopyranosyl (1-2)]-α-L-arabinopyranoside (**27**), and 26-*O*-β-d-glucopyranosylfurostanol-5,25(27)-diene-1β,3β,22β,26-tetraol-1-*O*-[α-l-rhamnopyranosyl-(1-2)]-α-l-arabinopyranoside (28) [13]. The structures are listed Figure 6.

### 2.3. Lignans

**Figure 7:**
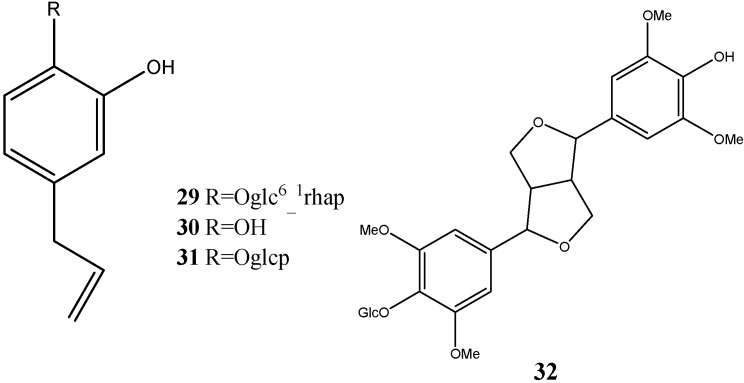
Structures of lignans.

Lignans have been isolated from dragon’s blood, namely 3,4-dihydroxyphenyl allyl-4-*O*-[α-l-rhamnopyranosyl (1-6)]-β-d-glucopyranoside (**29**), 3,4-dihydroxyallylbenzene (**30**), 3,4-dihydroxyphenyl 4-*O*-β-d glucopyranoside (**31**) and eleutheroside B (**32**) (Figure 7) [12,14].

### 2.4. Phenolic Constituents

**Figure 8:**
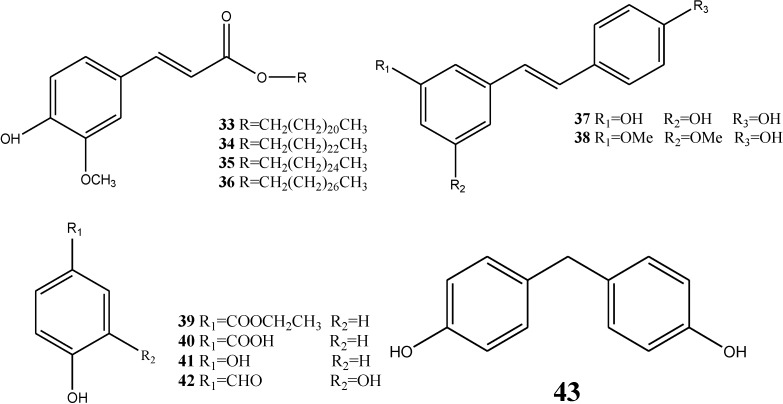
Structures of phenolic constituents.

Phenolic esters, esters and chlorine compounds have been isolated from dragon’s blood, namely ferulic acid docosyl ester (**33**), ferulic acid tetracosyl ester (**34**), ferulic acid hexacosyl ester (**35**), erulic acid octacosyl ester (**36**), resveratrol (**37**), pterostilbene (**38**), ethylparaben (**39**), *m*-hydroxybenzoic acid (**40**), hydroquinone (**41**), protocatechualdehyde (**42**), and 2-hydroxyphenylmethane (**43**) [12,14]. The structures are illustrated in Figure 8.

### 2.5. Other Ingredients

Other compounds, not listed above, have been isolated from dragon’s blood. Using GC-MS, the compounds that can be identified from the petroleum ether extract of dragon’s blood include alkanes (heneicosanoic, behenic, tricosane, tetracosane, pentacosane, hexacosane, heptacosane), olefins (trideca-1,17-diene), acids (palmitic acid, decanoic acid, palmitic acid, heptadecanoic acid, 3,4-dimethylcinnamic acid, 11,14-octadecadienoic acid, 2-cyclopentenetridecanoic acid), esters (pentadecylethyl carbonate, ethyl palmitate, 11,14-nonadecenoic dienoate, methyl nonadecene, ethyl stearate, ethyl eicosanoate, ethyl benzene dicarboxylate, heptadecyltetramethylammonium oleate), and aromatic constituents (1,2,4,6-tetramethylbenzene, γ-murrolene, α-caryophyllene, 4,5,9,10- tetrahydroisolongifolene, farnesene, acacia ketone) [15].

## 3. Pharmacological Effects

### 3.1. Invigorated Blood Circulation

Huang *et al.* [16] reported that dragon’s blood can regulating hemorrheology and inhibit experimental arterial thrombosis by facilitating blood circulation and dispersing blood stasis. Dragon's blood had no significant effect on the hemorrheology of normal rabbits. In a rabbit model of acute blood stasis induced by dextran, dragon’s blood significantly decreased the viscosity of whole blood as well as plasma, reduced the hematocrit, and accelerated the erythrocyte electrophoresis time. Dragon’s blood exhibited a significant inhibitory effect on experimental arterial thrombosis in mice. After administration of dragon’s blood, rabbits’ euglobulin lysis time (ELT) was shortened. Due to the inverse relationship between ELT and fibrinolytic activity, it was deduced that dragon’s blood could increase fibrinoclase levels and enhance its activity. The inhibition of the cyclooxygenase and TXA_2_ synthetase and the promotion of PGI_2_ synthase may be one of the mechanisms by which dragon’s blood affects blood circulation. Similar results were reported by Huang *et al.* [17], who also verified that total flavonoids were responsible for the therapeutic effects of dragon’s blood. The total flavonoids of dragon’s blood (TFDB) have a certain effect of increasing cerebral blood flow and reducing the brain water content in acute ischemic rat. The effect of TFDB on platelet aggregation, the thrombosis formation and the myocardial ischemia have also been investigated [18]. The results demonstrated that TFDB inhibits adenosine diphosphate (ADP)-induced platelet aggregation in rats and platelet-activating factor (PAF)-induced platelet aggregation in rabbits. And TFDB also can inhibit the formation of deep vein thrombosis in experimental rat and the area of acute myocardial ischemia caused by coronary artery ligation. This study proved that TFDB exerted the effects of facilitating blood circulation and dispersing blood stasis via inhibition of platelet aggregation and thrombosis formation. Dragon’s blood could significantly inhibit rabbit’s platelet aggregation induced by arachidonic acid *in vivo* and *in vitro*, and significantly inhibited thrombus formation induced by collagen-adrenaline *in vivo* in mice [19]. Furthermore, the *in vivo* platelet inhibition test, performed with the dose of 200 mg/kg on rats, demonstrated that the peak inhibitory effect of dragon’s blood was 35.8%, compared with the control group. With the *in vitro* concentrations of 0.2, 0.4 and 0.8 mg/mL, dragon’s blood exerted significant inhibition of aggregation by 18.7%, 20.0%, and 61.6%, respectively. The ethyl acetate fraction from dragon’s blood contains pharmacologically effective compounds with antithrombotic effects, partially improving platelet function and anticoagulation activity [20].

### 3.2. Hemostasis

Dragon’s blood has a hemostatic effect, and it is effective for both external and internal injuries. Nong *et al.* did an experiment to determine the clotting time of mouse blood using a capillary method, which proved that dragon’s blood can shorten the clotting time of mouse blood. As for the rabbits’ plasma recalcification time and prothrombin time, measurements showed that dragon’s blood could shorten the recalcification time, but has no significantly effect on the rabbits’ prothrombin time. So we can conclude that its hemostatic effect is related to the clotting factor of an endogenous coagulation system [21]. The experiment indicates dragon’s blood can significantly reduce the clotting time of mice blood [19]. Three compounds were isolated—3,4'-dihydroxy-5-methoxystilbene, cochinchinenin A and loureirin B—that have an inhibitory effect on ADP-induced platelet aggregation *in vitro* [22]. These effects have been confirmed by other experiments [23].

### 3.3. Lowering Blood Sugar, Blood Lipids

The effects of dragon’s blood on blood glucose, plasma insulin and blood lipids of mice has been studied [24]. The experiment involves administering dragon’s blood to mice in a hyperglycemia physiological model (caused by glucose or adrenaline) and in diabetic models (caused by alloxan), then measuring blood glucose, plasma insulin, total cholesterol, triglycerides, and high-density lipoprotein cholesterol. The results showed that dragon’s blood can significantly decrease blood glucose levels in hyperglycemic mice (glucose and adrenaline-induced); improve glucose tolerance; reduce the fasting blood glucose level in diabetic mice (alloxan-induced); increase insulin secretion in normal and diabetic mice; lipid-lowering effect, but has no obvious effect on the fasting blood glucose of normal mice. This indicates that dragon’s blood has a good therapeutic effect on animals with hyperglycemia from various causes, and has a certain lipid-lowering effect. We hypothesize that increasing insulin secretion is a primary mechanism by which dragon’s blood exerts is hypoglycemic effects. It has been proved that dragon’s blood extract has a good therapeutic effect on mice in a diabetic model (caused by alloxan). It can increase serum insulin levels, and its active components can be absorbed and enter the blood to function. Dragon's blood’s active constituents have a noncompetitive, inhibitory effect on α-glycosidase. The half-inhibitory concentration is 0.152 μg/mL, which is close to half of that of the crude drugs [25]. In addition, when this extract is administered orally to mice in effective amounts (100, 300, 500 mg/kg), it can significantly inhibit in a dose-dependent way the increase glucose levels after glucose loading. These results show that dragon’s blood extracts produce hypoglycemic effects by inhibiting the intestinal absorption of carbohydrates to reduce the postprandial rise in blood sugar. In this way, dragon’s blood plays a role in the treatment of diabetes.

### 3.4. Anti-Bacterial Effects

At a concentration of 0.25 mg/mL, dragon’s blood can inhibit the growth of *Staphylococcus aureus, Diphtheria bacilli* and *Bacillus anthracis*; when the concentration reaches 50 mg/mL, it can inhibit the growth of *Candida albicans*, *Cryptococcus neoformans* and the Shen Keshi-borne fungus *Sporothrix* [26]. Chen *et al.* [27] affirmed the antibacterial effect of dragon’s blood based on experimental results: the minimum inhibitory concentration (MIC) of dragon’s blood against common bacteria, such as *Staphylococcus aureus*, *S. lemon*, *S. diphtheria* was 3.12 mg/kg. As for *Escherichia coli*, *Salmonella typhi*, *Pseudomonas aeruginosa, Nessler cocci* and *Shigella flexneri*, the MIC values were not more than 50 mg/kg. Wang *et al.* [28] investigated the anti-fungal activity of three phenolic ingredients in dragon’s blood. Ethyl paraben has a strong anti-fungal and antiseptic effect, while 7,4-dihydroxyflavan and 7-hydroxy-4-methoxyflavan have significant activity towards the main fungi in cochinchinenin trees’ stems, so it is considered that these phenolic compounds are the plant defenses that *Dracaena* produces after fungal infection, and these compounds have potential for use in anti-bacterial and anti-inflammatory pharmaceuticals. It has been reported that new flavonoid derivatives 6,7- and (2S)-4',7-dihydroxy-8-methylflavan were are very effective against *H. pylori*, with MIC values of 29.5, 29.5, and 31.3 µM, respectively [29]. Pterostilbene can also inhibit the growth of *Trichophyton rubrum, T. mentogrophtes*, *Candida albican*, *C. parapsilosis*, *Cryptococcus neoformans*, and *Aspergrillus fumigates* [30]. 

### 3.5. Enhancing Immune Function

Mice were given 0.072 g/kg dragon’s blood and the results showed there was a significant increase in spleen weight in female mice. Microscopic examination showed that the spleen follicular germinal center was enlarged, and that the medullary cord plasma cells, multicore giant cells and reticular cells were significantly increased. This indicates that dragon’s blood can enhance the performance of the immune system [31].

### 3.6. Promoting Skin Repair

The role that dragon’s blood plays in promoting skin wound repair in tissue engineering has been observed [32]. The scientists planted human fibroblasts into collagen sponge, and grew human keratinocytes on the surface, whereby the tissue engineering skin was built; they then transplanted it into skin wounds on nude mice. The rats were randomly divided into four groups. Two days after transplantation, dragon’s blood was applied externally to one group, administered orally to one group, and given both externally and orally to one group, and while the fourth group received no treatment as a blank control. Nine days after the tissue-engineered skin grafts were applied, samples were collected and analyzed, recording the epidermal thickness of the local wound, the expression of laminin (Ln), the amount of collagen I in dermal layer, and the extent of capillary hyperplasia. The results showed dragon’s blood can promote the development of transplanted epidermis in tissue-engineered skin, the proliferation of capillaries in the dermis, and can enhance Ln and collagen I secretion. In addition, dragon’s blood is most effective when used orally and externally. The scientists also explored the effects that different active sites of dragon’s blood on fibroblast growth and secretion in cultured fetal rat skin. They found that the active parts of dragon’s blood ethyl acetate extract have a mild inhibitory effect on fetal mouse fibroblast cell growth at low concentrations, and that the growth-promoting effect increases with the concentration. One g/L active parts of dragon’s blood ethyl acetate extract has the best growth-promoting effect on fetal rat skin fibroblasts, and can also promote the secretion of Ln and collagen I [33].

### 3.7. Anti-Spasmodic Effect

Dragon’s blood, administered at a rate of 1.8 g/kg can significantly antagonize mouse uterine smooth muscle contraction caused by diethylstilbestrol.

### 3.8. Anti-Inflammatory Effects

Dragon’s blood applied externally to the skin can significantly inhibit croton oil-induced inflammation in mouse ears; it can also reduce carrageenan-induced rats paw edema and acetic acid-induced rat peritoneal capillary permeability. These results prove that dragon’s blood has anti-inflammatory effects. In addition, Lin’s experiments [29] proved that 2 g/kg dragon’s blood administered by gavage to mice can effectively inhibit xylene-induced mice ear inflammation. Dragon’s blood with 20% concentration used externally can accelerate and promote wound healing in rabbit’s burn-induced inflammation [31].

### 3.9. Analgesic Effects

Dragon’s blood is often used clinically as an analgesic. Xiang *et al.* found that administering dragon’s blood 1.72 g/kg and 3.44 g/kg for five consecutive days significantly inhibit mice’s acetic acid-induced writhing [34]. Cochinchinenin B inhibits capsaicin (CAP) starting current (ICAP) of the dorsal root ganglia in isolated mice [35]. This inhibition was reversible, non-competitive, and neither voltage- nor agonist-dependent. Intracellular cochinchinenin B does not alter the inhibitory effect of ICAP, indicating that the binding sites of the channel are extracellular. In addition, cochinchinenin B can inhibit the depolarization caused CAP under inhibited current clamp. This suggests that it may be possible to develop cochinchinenin B derived from dragon’s blood as an analgesic.

### 3.10. Anti-Diabetic Effect

The active fraction extracted from dragon’s blood displayed an inhibitory effect on α-glucosidase activity with an IC_50_ of 0.152 mg/mL, which is nearly half the activity of crude material. Its inhibition on α-glucosidase was noncompetitive. In addition, when this fraction was orally administered to mice dosed with acarbose (20 mg/kg), the active fraction (100, 300, 500 mg/kg) significantly suppressed any increase of blood glucose levels after sucrose loading in a dose-dependent manner. These results suggest that this extract from dragon’s blood exerts an anti-diabetic effect by suppressing intestinal carbohydrate absorption, thereby reducing the postprandial increase of blood glucose [36].

### 3.11. Anti-Tumor Effects

Cholest-4α-methyl-7-en-3β-ol has potent inhibitory activity against PC_12_ tumors with a ratio of 0.5043 (10 μg/mL). The synthesized derivatives were tested on human cancer cell lines including colon (HCT-8), liver (BEL-7402) and nasopharyngeal cancer (KB) cells. The results showed that cholest-4α-methyl-8-en-3β,7α-diol 6a inhibits KB cells significantly with an IC_50_ value of 1.32 × 10^−9^ μg/mL. In addition, the cytotoxic properties of this compound against HCT-8 and BEL-7402 cells are excellent, with an IC_50_ of 1.2 μg/mL [37].

## 4. Toxicology Studies

To test long-term toxicity, rabbits were given Guangxi dragon’s blood at rates of 3 g/kg body weight and 1.5 g/kg body weight, once daily, for 90 days. The dragon’s blood did not cause changes in the animal’s pathological state, and had no significant effect on blood erythrocytes, leukocytes number, alanine aminotransferase, urea nitrogen, or weight. There was no functional damage to the liver or kidney. In the pathological examination under optical microscope, except for some expansion of the tiny blood vessels between myocardial cells, there was no damage to liver, lung, kidney, intestine or the adrenal glands. Gavaging the mice with the clinical daily dose of Guangxi dragon’s blood (0.072 g/kg) 150 times continuously for 30 days caused no toxic reactions [38].

## 5. Clinical Applications

### 5.1. Internal Medicine

#### 5.1.1. Coronary Heart Disease

The treatment of coronary heart disease with Chinese traditional medicine that contains dragon’s blood component (single-serving alone/compound) has been shown to have significant beneficial effects. The dragon’s blood capsules were served combined with Western conventional treatment (thrombolysis, oral isosorbide dinitrate, aspirin, enalapril and other medicined) to treat 36 patients who had had acute myocardial infarction (AMI) for 3–4 weeks; a comparison group with 34 patients was treated only with conventional therapy. The results showed that the treatment group has better improvement of hemorheology indicators, and the treatment was more effective (*p* < 0.01) [39]. An experiment using random, double-blind, positive controlled, multi-center clinical scientific research methods, evaluated the efficacy and safety of dragon's blood capsules to treat stable angina (blood stasis syndrome, in TCM terms). In a total of 418 patients, 314 in the experimental group were administered dragon's blood capsules, while 104 cases in the control group, took Danshen capsules. The results showed that the angina and ECG indicators improved significantly more in the experimental group than in the control group (*p* < 0.05), and no adverse reactions were observed [40]. In another experiment, three patients with coronary heart disease and arrhythmias continuously took “Rainforest Brand” dragon’s blood powder for two months; at the end of the experimental period, symptoms were either relieved or improved, and the review ECGs were normal [41]. In another experiment, Zhang *et al.* [42] showed that the myocardial ischemia of the rats was improved by high and medium doses of dragon’s blood. The formation of the thrombus was controlled and the coagulation time was shortened effectively. These results suggest the dragon’s blood may act on the microcirculation arteries, dissolving clots, improving blood circulation, and softening blood vessels, so it can reverse the hardening and narrowing of small arteries, thus relieving the clinical symptoms of coronary heart disease.

#### 5.1.2. Upper Gastrointestinal Bleeding

“Rainforest brand” dragon’s blood was used to treat 42 cases of acute upper gastrointestinal bleeding patients. Of these, 25 cases showed remarkable effects, while in 17 cases treatment was effective and no side effects were found. The report indicates that in the treatment of upper gastrointestinal tract bleeding, dragon’s blood has the advantages of being fast, safe, convenient, and economical [43].

#### 5.1.3. Peptic Ulcer

Peptic ulcer is mainly due to the loss of balance between the damaging factors of the gastroduodenal mucosa and partial duodenal mucosa with the mucosal protective factors. Omeprazole and amoxicillin plus dragon’s blood were used in 32 patients with peptic ulcer (treatment group), and compared with 40 cases in the control group (conventional Western medicine) for 4 weeks. The results showed that when dragon’s blood was used, the pain relief time was significantly shortened, hemostasis quicker, ulcer healing relatively quicker, and negative rate of Hp was higher [44].

#### 5.1.4. Chronic Nonspecific Ulcerative Colitis (Chronic Colitis)

Single-dose “Rainforest brand” dragon’s blood in warm water enemas were used to treat chronic non-specific colitis, and had a good effect [45]. In a total of 12 patients, six cases were cured, in five cases it had a marked effect, and one was effective. Oral administration of “Spruce brand” dragon’s blood capsules and dragon’s blood powder enemas with 5-aminosalicylic acid (SASP) (oral administration and enema) was used to treat 162 cases of ulcerative colitis [46]. The results showed the complete remission rate and total efficiency were similar, but the adverse reactions of dragon’s blood were little. These results indicate that in treatment of chronic colitis, dragon’s blood is reliable, and has few adverse reactions. In an experiment with 40 cases of UC, they were divided into a treatment group (20 cases) and a control group (20 cases) [47]. Both groups were given SASP per-oral administration and retention-enema. In addition, the treatment group was given dragon’s blood orally and by retention-enema. The result showed that the short-term curative rate, the total effective rate and the recurrence rate were 60%, 95% and 15.8%, respectively, in the treatment group, and 20%, 65% and 61.5%, respectively, in the control group. There was a significant difference between the two groups (*p* < 0.05). These results indicate that integrative therapy using dragon’s blood with Western pharmaceuticals can greatly promote the relief and reduce the recurrence of UC.

#### 5.1.5. Type II Diabetes and Its Complications

The “Rainforest brand” dragon’s blood powder was used to treat 36 cases of hyperlipidemia in patients with Type II diabetes [48]. After three cycles of treatment (90 days), the total cholesterol, triglycerides and other indicators showed a significant decrease. The patients reported no particular discomfort or other side effects, indicating that the drug was safe and reliable. In another experiment, diabetic patients treated their foot ulcers topically with dragon’s blood powder and oral administration of dragon’s blood capsules combined with conventional Western medicine. The total effective rate and ulcer healing time in the treatment group were significantly better than in the control group. From the Chinese medicine perspective, these two effects are related to the blood circulating effect and the myogenic and rot removing effect. According to modern pharmacological research, it is related to the inhibition of platelet aggregation, expansion of blood vessels, increase in blood flow, and killing of bacteria [49,50,51].

#### 5.1.6. Hyperthyroidism

Hyperthyroidism is an autoimmune deficiency disease, and pharmacological studies suggest that dragon’s blood adjusts the immune system in two ways. In one study, a total of 64 hyperthyroidism patients were divided into two groups [52]. The experimental group (34 cases) took “Rainforest brand” dragon’s blood capsules, either orally or applied them externally, while the control group (30 cases) used methimazole treatment. The result showed no difference in effect between the two groups, but the experimental group didn’t suffer from the side effects of methimazole. Further study is needed.

### 5.2. Gynecology

#### 5.2.1. Cervical Erosion

Yunnan dragon’s blood using externally was used to treat 58 cervical erosion patients [53]. The result showed significant effect, after a short course of treatment with no side effects.

#### 5.2.2. Uterine Fibroids

Uterine fibroids is the most common form of female genital benign tumor. “East Dragons brand” dragon’s blood capsules were used to treat 36 uterine fibroid patients; of these, 29 cases showed a significant effect (80.5%), five cases showed some effect (13.8%), and two cases showed no effect (5.6%), so the total efficiency was 94.3% [54]. Dragon’s blood can reduce the increased amount of menstrual bleeding caused by uterine fibroids and also can shrink fibroids. In these experiments, it was effective, safe and without toxic side effects.

#### 5.2.3. Uterine Bleeding

40 dysfunctional uterine bleeding cases were treated with dragon’s blood capsules, taken orally [55]. Results showed that, when treating menorrhagia and dysmenorrheal, a short course of treatment can have a significant, beneficial effect.

#### 5.2.4. Ovarian Cysts

Forty ovarian cysts patients were divided into two groups according to age. Results showed that dragon’s blood helped all patients, but was more effective for younger patients [56].

### 5.3. Anorectal Disease

#### 5.3.1. Hemorrhoids

Various types of acute external hemorrhoids 119 cases were treated orally and topically with “Rainforest brand” dragon’s blood [57]. It was especially effective for thrombotic external hemorrhoids, inflammatory hemorrhoids and varicose vein swelling; the swelling could be significantly reduced in one day. There were no adverse reactions; no need to treat with antibiotics, and it was easy for patients to accept achieved satisfactory results.

#### 5.3.2. Anal Fissures

Lei *et al.* [58] used dragon's blood capsules to treat early fissure and chronic anal fissure in 130 post-operative cases. All patients experienced wound healing, and cure of anal pain, bleeding and other symptoms in 6–15 days.

### 5.4. ENT Diseases

#### 5.4.1. Epistaxis

One hundred and forty-four epistaxis patients were treated with dragon’s blood capsules administrated orally and topically, while dicynone and bleeding aromatic acid infusion were used to treat another 144 cases in a control group [59]. The effective rate in the treatment group was 86.81%, while the effective rate in the control group was 77.08%. There were significant differences between the two groups (*p* < 0.05).

#### 5.4.2. Traumatic Tympanic Membrane

The clinical efficacy of external application of Yunnan dragon’s blood capsules powder to treat traumatic tympanic membrane perforation was observed [60]. Two hundred and six cases of (ear) eardrum trauma patients were randomly divided into two groups; the control group used eardrum-shaped cotton pieces with 4% alcohol boric glycerin to fill up the perforated tympanic membrane and orally took antibiotics and vitamin C for 6 weeks, while the treatment group did the same, but also applied a cotton patch with dragon's blood powder on the outside of the ear. Healing time and condition of the tympanic membrane before and after treatment was recorded. The results showed that both the healing rate and the average healing time of the treatment group were better than the control group, so it can be concluded that dragon's blood powder can improve the cure rate and shorten the treatment time of traumatic tympanic membrane.

### 5.5. Other Uses

#### 5.5.1. Soft Tissue Injury

Dragon’s blood has a good therapeutic effect in soft tissue injury, as it has anti-inflammatory and analgesic effects, and can improve blood circulation and convergence sores. The treatment of 263 acute soft tissue injured patients with dragon's blood capsules (or enteric-coated tablets), taken orally, was reported [61]. The total effective rate was 98%, and the healing time was shortened. Another experiment reported that the treatment of soft tissue injury in 40 patients by oral administration of Guangxi dragon’s blood powder and external application of Guangxi dragon’s blood tincture; the total efficiency was 100% [62].

#### 5.5.2. Radioactive Moist Dermatitis

Tang *et al.* [63] discussed the therapeutic efficacy of dragon’s blood powder in treating radioactive moist dermatitis. In this study, 80 radiation dermatitis patients were randomly put to two groups; the experimental group was treated with dragon's blood powder, while the control group members were treated with MEBO. The results showed that the efficiency of experimental group was 97.5% while the control group was 82.5%. The difference was significant (*p* < 0. 01). Hou *et al.* [64] used dragon’s blood powder to treat 63 radiation moist dermatitis patients with nasopharyngeal carcinoma. Those receiving treatment healed significantly more quickly than the control group (using normal saline, dexamethasone, gentamicin). Also, the average interruption duration of radiotherapy was shorter than the control group.

#### 5.5.3. Burn

It is clinically proven that the “Rainforest brand” dragon’s blood from Yunnan has significant effects not only on superficial burns, but also on deep burns [65,66].

#### 5.5.4. Alopecia Areata

Alopecia areata is a locally patchy skin disease. Twenty patients were treated with dragon’s blood capsules from Yunnan Province orally, combined with external application of ginger. The total efficiency was 81% [67].

#### 5.5.5. Psoriasis

Psoriasis is a recurring, stubborn, intractable chronic inflammatory skin disease that has psychosomatic components or correspondences. It was reported, in one experiment with 60 patients treated by oral administration of dragon's blood capsules that treatment efficiency reached 89% [68].

#### 5.5.6. Pressure Sores

Bedridden patients often get pressure sores due to poor blood circulation, resulting in local skin rot and, often, infection. As a traditional Chinese medicine, dragon’s blood has the effect of circulating the blood, remove the rot and of myogenic, so when used outside, the effect was good.

#### 5.5.7. Stroke

In treatment of 21 post-stroke patients, the conventional therapy combined with oral administration of dragon’s blood capsules had a total effective rate of 90% [69]. There were obvious effects in restoring muscle strength, stabilizing blood pressure, lowering blood sugar and improving blood viscosity.

## 6. Conclusions

This review has summarized the botanical source, the phytochemisty, the pharmacological effects, the toxicology studies, and the clinical applications of the Chinese medicine dragon’s blood, which is extracted from *D. cochinchinensis*. The references are collected from 63 articles covering different fields in the research on dragon’s blood. The main chemical constituent of this kind of dragon’s blood is flavonoids, and this medicine has significant invigorating effects on blood circulation of hemostasis. Since the plant *D. cochinchinensis* is mainly distributed in China, and this domestic dragon’s blood has similar effects as the imported dragon’s blood which also can be found in Chinese market [70], future studies can be focused on the comparisons between these two medicines, especially on their chemical constitutes.
